# Double‐blind, placebo‐controlled study of lurasidone monotherapy for the treatment of bipolar I depression

**DOI:** 10.1111/pcn.13137

**Published:** 2020-09-24

**Authors:** Tadafumi Kato, Jun Ishigooka, Mari Miyajima, Kei Watabe, Tomohiro Fujimori, Takahiro Masuda, Teruhiko Higuchi, Eduard Vieta

**Affiliations:** ^1^ Department of Psychiatry Juntendo University Tokyo Japan; ^2^ Laboratory for Molecular Dynamics of Mental Disorders RIKEN Center for Brain Science Wako Japan; ^3^ Institute of CNS Pharmacology Tokyo Japan; ^4^ Sumitomo Dainippon Pharma Co., Ltd. Tokyo Japan; ^5^ Sunovion Pharmaceuticals Inc. Marlborough USA; ^6^ Japan Depression Center Tokyo Japan; ^7^ The National Center of Neurology and Psychiatry Kodaira Japan; ^8^ Bipolar and Depressive Disorders Unit, Hospital Clinic, Institute of Neurosciences University of Barcelona, IDIBAPS, CIBERSAM Barcelona Spain

**Keywords:** antipsychotic agents, bipolar disorder, depressive disorder, lurasidone hydrochloride

## Abstract

**Aim:**

Previous studies conducted primarily in the USA and Europe have demonstrated the efficacy and safety of lurasidone 20–120 mg/day for the treatment of bipolar I depression. The aim of the current study was to evaluate the efficacy and safety of lurasidone monotherapy for the treatment of bipolar I depression among patients from diverse ethnic backgrounds, including those from Japan.

**Methods:**

Patients were randomly assigned to double‐blind treatment for 6 weeks with lurasidone, 20–60 mg/day (*n* = 184) or 80–120 mg/day (*n* = 169), or placebo (*n* = 172). The primary end‐point was change from baseline to Week 6 on the Montgomery–Åsberg Depression Rating Scale (MADRS).

**Results:**

Lurasidone treatment significantly reduced mean MADRS total scores from baseline to Week 6 for the 20–60‐mg/day group (−13.6; adjusted *P* = 0.007; effect size = 0.33), but not for the 80–120‐mg/day group (−12.6; adjusted *P* = 0.057; effect size = 0.22) compared with placebo (−10.6). Treatment with lurasidone 20–60 mg/day also improved MADRS response rates, functional impairment, and anxiety symptoms. The most common adverse events associated with lurasidone were akathisia and nausea. Lurasidone treatments were associated with minimal changes in weight, lipids, and measures of glycemic control.

**Conclusion:**

Monotherapy with once daily doses of lurasidone 20–60 mg, but not 80–120 mg, significantly reduced depressive symptoms and improved functioning in patients with bipolar I depression. Results overall were consistent with previous studies, suggesting that lurasidone 20–60 mg/day is effective and safe in diverse ethnic populations, including Japanese.

Bipolar disorder is a chronic psychiatric disorder with an estimated lifetime prevalence worldwide ranging from 0.2% to 1.0%.^1^, ^2^ It is ranked among the top 20 causes of disability worldwide and among the top 10 causes of disability in developed countries.^3^ The impairment in social and occupational functioning due to bipolar disorder is extensive. The impact on work functioning is observed in lost days from work, loss of employment, and difficulty in regaining employment.^4^, ^5^ The overall health‐related quality of life found in individuals with bipolar disorder is impaired to a level comparable with other serious and chronic medical illnesses.^6^ Bipolar disorder has been shown to reduce an individual's expected life span by approximately 9 years.^7^ This is a result of a number of factors, including a higher rate of comorbid medical conditions (such as obesity, diabetes, and cardiovascular disease) and suicide.^8^, ^9^, ^10^ The annual economic burden of bipolar disorder in the USA has been estimated to be about $31 billion in direct costs and an additional $120 billion in indirect costs.^11^ In Japan, many individuals with bipolar disorder report a time lag of many years before receiving an accurate diagnosis and also report inability to work or study.^12^

Though episodes of mania or hypomania alternating with depressive episodes are the traditional view of bipolar disorder, studies have found that major depressive episodes are more common than manic episodes over the course of the illness.^13^, ^14^ However, treatment options are limited for depressive episodes compared to manic episodes; for example, in Japan, olanzapine and quetiapine extended release (XR) are the only approved treatments for bipolar depression before the approval of lurasidone in 2020.^15^, ^16^

Lurasidone is an atypical antipsychotic with high affinity for D_2_, 5‐HT_7_, and 5‐HT_2A_ receptors (antagonist), moderate affinity for 5‐HT_1A_ receptor (partial agonist), and no clinically relevant affinity for receptors such as histamine H_1_ and M_1_ receptors (IC_50_, >1000 nM), or the 5‐HT_2C_ receptor (Ki, 415 nM).^17^ Lurasidone therefore shares the D_2_ and 5‐HT_2A_ antagonist characteristics of second‐generation antipsychotics that are hypothesized to contribute to antipsychotic efficacy, with the 5‐HT_2A_ antagonism potentially limiting D_2_‐antagonist‐induced extrapyramidal adverse effects and prolactin elevation. The high antagonist activity of lurasidone at the 5‐HT_7_ receptor differentiates it from other second‐generation antipsychotics. This activity has been implicated in antidepressant like effect in animal models.^18^ Consistent with this, lurasidone has demonstrated efficacy in animal models of depression.^19^

The efficacy of lurasidone for the acute treatment of bipolar I depression has been established in two 6‐week randomized, double‐blind, placebo‐controlled studies. These included studies of lurasidone as a monotherapy^20^ and as an adjunctive therapy with lithium or valproate.^21^ In both studies, lurasidone was found to reduce primary measures of depressive symptoms to a significantly greater degree than placebo with minimal changes in weight or metabolic parameters. Lurasidone has been approved for bipolar I depression in the USA and several other countries and is recommended as a first‐line treatment for bipolar I depression in international guidelines.^22^, ^23^, ^24^

Despite the two positive trials of lurasidone as a monotherapy or adjunctive therapy for bipolar depression,^20^, ^21^ the generalizability of the results to diverse ethnic populations is not clear. Although both trials were multicenter international studies, recruitment in these studies was limited to sites in the USA, Europe, and South Africa. Of those randomized, 9.3% and 23.5% of the treatment samples were from an Asian background in the monotherapy and adjunctive therapy studies, respectively.

The goal of the current study was to further evaluate the efficacy and safety of lurasidone as a monotherapy for bipolar I depression with recruitment across a range of international sites, including Japan.

## Methods

### Patients

This study enrolled male and female outpatients aged 18–74 years who were currently experiencing a major depressive episode of at least 4 weeks but less than 12 months in duration, and were diagnosed with bipolar I disorder, utilizing DSM‐IV‐TR criteria, and including a history of at least one bipolar manic or mixed manic episode. A history of rapid cycling was permitted if episode frequency in the 12 months prior to screening was ≥4 episodes but <8 episodes. Diagnosis was determined using the Mini‐International Neuropsychiatric Interview.^25^ To be included, patients also needed to have a Montgomery–Åsberg Depression Rating Scale (MADRS)^26^ score ≥ 20 and a Young Mania Rating Scale (YMRS)^27^ score ≤ 12 at both screening and baseline.

Patients were excluded if they had current psychotic features (a past history was not exclusionary) or had another Axis I or Axis II disorder other than bipolar disorder that was the focus of treatment received in the 3 months prior to screening. Also excluded were patients: who scored ≥4 on MADRS Item 10 (suicidal thoughts) at screening or baseline; who had a history of nonresponse to an adequate (6‐week) trial of three or more antidepressants (with or without mood stabilizers) during the current depressive episode; who had been hospitalized for a manic or mixed episode within 60 days of screening; and who responded *Yes* to the Columbia Suicide Severity Rating Scale (C‐SSRS)^28^ Item 4 (active suicidal ideation with some intent to act, without specific plan) or Item 5 (active suicidal ideation with specific plan and intent) at screening (within 6 months prior to screening) or at baseline, or were otherwise judged to be an imminent risk of suicide or injury to self, others, or property.

An institutional review board at each investigational site reviewed and approved the study. The study was conducted in accordance with the International Conference on Harmonization Good Clinical Practices guidelines and with the ethical principles of the Declaration of Helsinki. Prior to enrollment, all patients reviewed and signed an informed consent document explaining study procedures and potential risks and authorizing publication. The study was monitored by an independent data and safety monitoring board throughout the study.

### Study design

This study was a randomized, double‐blind, placebo‐controlled, flexible‐dose, parallel‐group, monotherapy study of lurasidone. Patients were enrolled at 102 centers in eight countries. This included 55 centers in Japan, three centers in Lithuania, five centers in Malaysia, three centers in the Philippines, 19 centers in Russia, five centers in Slovakia, three centers in Taiwan, and nine centers in the Ukraine. The study was conducted from February 2014 to February 2017.

Following a washout period of 3 or more days (as needed), patients were randomly assigned in a 1:1:1 ratio via an interactive web response system to receive 6 weeks of lurasidone 20–60 mg/day (flexibly dosed), lurasidone 80–120 mg/day (flexibly dosed), or placebo. Study medication was provided in blister packs as either lurasidone 20 mg or identically matched placebo tablets. Lurasidone dosing was fixed at 20 mg/day for Days 1 to 7 in the 20–60‐mg/day treatment arm. Patients randomized to the lurasidone 80–120‐mg/day treatment arm were to receive 20 mg/day on Days 1 and 2, 40 mg/day on Days 3 and 4, 60 mg/day on Days 5 and 6, and 80 mg/day on Day 7. Thereafter, in both lurasidone groups, lurasidone was to be flexibly dosed starting on Day 8. At the scheduled visits at Week 1 or after, when no safety concerns were found and the Clinical Global Impression: Bipolar Version – Severity of Illness (CGI‐BP‐S^29^) Depression score was within the range of 5 (*markedly ill*) to 7 (*very severely ill*), the dose was to be increased by 20 mg/day. If any safety concerns were evident, the dose could be reduced by 20 mg/day at an unscheduled visit. Lurasidone (or placebo) was taken once daily, within 30 min after the evening meal.

### Concomitant medications

During the treatment period, lorazepam (≤2 mg/day) was permitted as needed for the treatment of anxiety, agitation, irritability, and related psychiatric symptoms, between screening and Week 3. Hypnotics were permitted between screening and Week 3 for insomnia. All other psychotropic medications were prohibited for the full treatment period. Other medications prohibited during the study were known CYP3A4 inhibitors and inducers as well as Chinese herbal medications. For patients treated with antiparkinson agents at screening, the medications were to be titrated down appropriately and terminated before the initiation of the study treatment. For all patients, if any extrapyramidal symptoms developed or worsened after the initiation of the study treatment, permitted antiparkinson medications included biperiden (≤16 mg), trihexyphenidyl (≤10 mg), benztropine (≤6 mg), and diphenhydramine (≤300 mg). For akathisia, propranolol (≤120 mg/day), amantadine (≤300 mg/day), or one of the allowable antiparkinson medications was permitted.

### Efficacy assessments

The primary efficacy end‐point was the mean change in the MADRS total score (range: 0–60) from baseline to Week 6. Certified raters conducted the MADRS assessment at each visit. Secondary efficacy assessments included the CGI‐BP‐S Depression score (range: 1–7), the Sheehan Disability Scale (SDS)^30^ total score (range: 0–30), the Hamilton Anxiety Rating Scale (HAM‐A^31^; range: 0–56), the YMRS (range: 0–60), MADRS response and remission rates, and time to response. The MADRS, CGI‐BP‐S, and YMRS were obtained at baseline and every week for 6 weeks; the HAM‐A and SDS were obtained at baseline and Week 6. Response was defined as a ≥50% reduction from baseline in the MADRS total score at Week 6; remission was defined as an MADRS total score of ≤12 at Week 6. Exploratory *post‐hoc* efficacy analysis was also conducted on the individual MADRS item scores and the MADRS‐6 subscale,^32^ which evaluates the ‘core depressive symptoms’ (as assessed by the MADRS items: apparent sadness, reported sadness, inner tension, lassitude, inability to feel, and pessimistic thoughts).

### Safety and tolerability evaluations

Safety and tolerability were assessed throughout the study by: the incidence and severity of treatment‐emergent adverse events (TEAEs); laboratory measures of prolactin, glucose metabolism, and lipid metabolism; vital signs; weight; and QTc interval determined from electrocardiography (ECG) measurements. In addition, the influence of treatment on extrapyramidal symptoms was evaluated, as evidenced by extrapyramidal adverse events, proportion of patients using concomitant antiparkinson drugs, and changes on the Drug‐Induced Extrapyramidal Symptoms Scale (DIEPSS).^33^ Treatment‐emergent mania was defined as a YMRS total score of ≥16 at any two consecutive post‐baseline visits, or at the final assessment, or any TEAEs related to mania symptoms. Suicidal ideation and behavior were evaluated with the C‐SSRS.

### Statistical analysis

The intent‐to‐treat population consisted of randomly assigned patients who received at least one dose of study medication and had baseline and at least one post‐baseline MADRS total score. A mixed‐effects model for repeated measures (MMRM) was used to analyze the primary efficacy variable (the change from baseline in MADRS total score at Week 6). The MMRM method included treatment, visit, pooled center, baseline MADRS total score, and treatment‐by‐visit interaction. An unstructured covariance matrix was used for the within‐patient correlation. For the primary efficacy variable, *P*‐values were adjusted for multiplicity (comparisons of each of the lurasidone groups to placebo) using a Hochberg procedure. The individual MADRS item scores, the MADRS‐6 subscale, and CGI‐BP‐S score were also analyzed using the MMRM approach. Effect sizes at Week 6 were calculated as the absolute value of the least square (LS) mean difference from placebo divided by the model estimate of the standard deviation (SD), with both the LS mean and estimated SD obtained from the MMRM analysis. Additional secondary efficacy measures (SDS, HAM‐A, YMRS) were examined using an analysis of covariance (ANCOVA) model. The ANCOVA model included treatment as a categorical factor, pooled center, and the score on the efficacy measure at baseline as a covariate. The response variable for the model was the change from baseline in the efficacy measure at Week 6 using last observation carried forward (LOCF). Logistic regression was used to evaluate the proportions of patients achieving both treatment response and symptom remission. For these analyses, predictor variables included treatment group, pooled study site, and the MADRS total score at baseline, all entered using the forced‐entry method. Number needed to treat (NNT) was calculated as the reciprocal of the difference in the proportion of responders in the lurasidone group and the proportion of responders in the placebo group. For time‐to‐response analysis, patients failing to achieve MADRS response were censored. Kaplan–Meier plots were displayed, and a log–rank test and a Cox regression model analysis were used for comparing the treatment groups.

The population for safety analyses included all patients who were randomized and received at least one dose of study medication. MMRM analyses were used to examine treatment group differences in the change from baseline in the DIEPSS total score (excluding overall severity; range: 0–32). The numbers and percentages of patients with TEAEs, treatment‐emergent mania, and concomitant use of antiparkinson medication were summarized by treatment groups. Relevant summary statistics for laboratory tests, vital signs, bodyweights, and 12‐lead ECG parameters were calculated by treatment group. A rank ANCOVA method with adjustments for baseline values was applied to change from baseline to Week 6 (LOCF) in serum prolactin, blood glucose, HbA1c (National Glycohemoglobin Standardization Program), total cholesterol, LDL cholesterol, HDL cholesterol, triglycerides, weight, and body mass index for comparison between each lurasidone group and the placebo group. There was no multiplicity adjustment for the secondary efficacy analyses or safety analyses.

Sample size for the primary efficacy analysis was determined by a Monte‐Carlo simulation using SAS Version 9.2 (SAS Institute, Cary, NC, USA). A common effect size of 0.35 (i.e., an intergroup difference in change from baseline of 3.5 with an SD of 10) in the MADRS total score for both lurasidone groups over the placebo group was used for estimating the sample size. This resulted in a sample size of 161 per group for a total of 483 patients to yield power (probability of rejecting two null hypotheses) of 80%, with a 1‐sided 2.5% significance level using Hochberg procedure for multiplicity adjustment. To account for attrition (patients randomized who provide no post‐baseline MADRS total scores), the sample size was planned to be 501 patients or 167 patients per treatment group. The conservative estimate for assumed effect size 0.35 was made because of the limited number of placebo‐controlled clinical studies in patients with depressive symptoms associated with bipolar disorder in Japan or Asia where a portion of the current sample was recruited.

## Results

### Patients and disposition

**Fig. 1:**
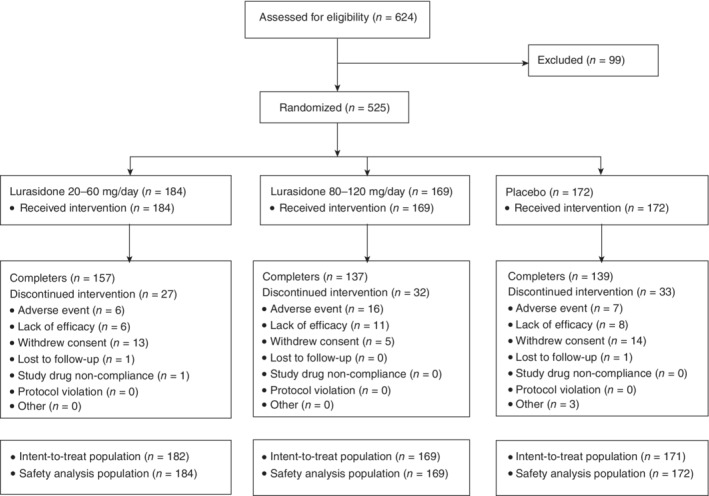
Patient disposition.

A total of 624 patients were screened for the study; 525 were randomly assigned to 6 weeks of double‐blind treatment (Fig. 1) and all of these received at least one dose of study medication (safety population). Three patients were excluded from the intent‐to‐treat population because of lack of at least one post‐baseline MADRS total score, resulting in an analysis sample of 182 patients in the lurasidone 20–60‐mg/day group, 169 in the lurasidone 80–120‐mg/day group, and 171 in the placebo group. Baseline demographic and clinical characteristics were similar among the three treatment groups (Table 1). There were 178 patients (34.1% of the study population) who received treatment in Japan. Study completion rates were 85.3% for the lurasidone 20–60‐mg/day group, 81.1% for the lurasidone 80–120‐mg/day group, and 80.8% for the placebo group (Fig. 1).

**Table 1:** Baseline characteristics (intent‐to‐treat population)

	Lurasidone	Lurasidone	Placebo
	20–60 mg/day	80–120 mg/day	
	(*n* = 182)	(*n* = 169)	(*n* = 171)
Demographics and clinical characteristics			
Female sex, *n* (%)	95 (52.2%)	88 (52.1%)	94 (55.0%)
Age (years), mean (SD)	42.6 (12.9)	43.2 (12.8)	41.3 (12.6)
Race, *n* (%)			
White	105 (57.7%)	103 (60.9%)	94 (55.0%)
Asian	77 (42.3%)	66 (39.1%)	77 (45.0%)
Country, *n* (%)			
Japan	65 (35.7%)	53 (31.4%)	60 (35.1%)
Malaysia	6 (3.3%)	6 (3.6%)	7 (4.1%)
Philippines	3 (1.6%)	3 (1.8%)	5 (2.9%)
Russia	51 (28.0%)	50 (29.6%)	44 (25.7%)
Taiwan	3 (1.6%)	4 (2.4%)	5 (2.9%)
Ukraine	43 (23.6%)	45 (26.6%)	43 (25.1%)
Lithuania	4 (2.2%)	4 (2.4%)	2 (1.2%)
Slovakia	7 (3.8%)	4 (2.4%)	5 (2.9%)
Duration of bipolar I disorder (years), mean (SD)	12.1 (10.5)	12.4 (10.7)	12.1 (9.7)
With rapid cycling, *n* (%)	19 (10.4%)	19 (11.2%)	16 (9.4%)
Efficacy measures			
MADRS total score, mean (SD)	30.6 (5.6)	30.8 (5.1)	30.9 (5.4)
CGI‐BP‐S Depression score, mean (SD)	4.57 (0.70)	4.58 (0.60)	4.60 (0.69)
SDS total score,^†^ mean (SD)	29.4 (5.3)	19.8 (5.6)	19.9 (5.2)
HAM‐A total score, mean (SD)	17.6 (7.2)	16.7 (6.2)	17.1 (6.6)
YMRS total score, mean (SD)	2.92 (2.49)	2.67 (2.29)	2.62 (2.23)
Safety measure			
DIEPSS total score (excluding overall severity), mean (SD)	0.41 (1.01)	0.39 (0.83)	0.36 (0.93)

^†^ Number of patients: lurasidone 20–60 mg/day = 154; lurasidone 80–120 mg/day = 127; placebo = 141.

CGI‐BP‐S, Clinical Global Impression, Bipolar Version ‐Severity of Illness; DIEPSS, Drug‐Induced Extrapyramidal Symptoms Scale; HAM‐A, Hamilton Anxiety Rating Scale; MADRS, Montgomery–Åsberg Depression Rating Scale; SDS, Sheehan Disability Scale; YMRS, Young Mania Rating Scale.

The mean (±SD) daily dose of lurasidone during the study was 36.2 ± 12.0 mg in the 20–60‐mg/day group and 85.4 ± 14.2 mg in the 80–120‐mg/day group. In the 20–60‐mg/day group, the percent of patients utilizing a modal dose of 20 mg, 40 mg, and 60 mg was 36.8%, 23.1%, and 40.1%, respectively. In the 80–120‐mg/day group, the percent of patients utilizing a modal dose of 80 mg, 100 mg, and 120 mg was 41.4%, 26.0%, and 30.2%, respectively. Use of lorazepam as needed was reported for 15.8% of patients in the lurasidone 20–60‐mg/day group, 17.2% in the lurasidone 80–120‐mg/day group, and 18.0% in the placebo group. Use of hypnotics as needed was reported for 24.5% of patients in the lurasidone 20–60‐mg/day group, 26.6% in the lurasidone 80–120‐mg/day group, and 28.5% in the placebo group.

### Efficacy

**Fig. 2:**
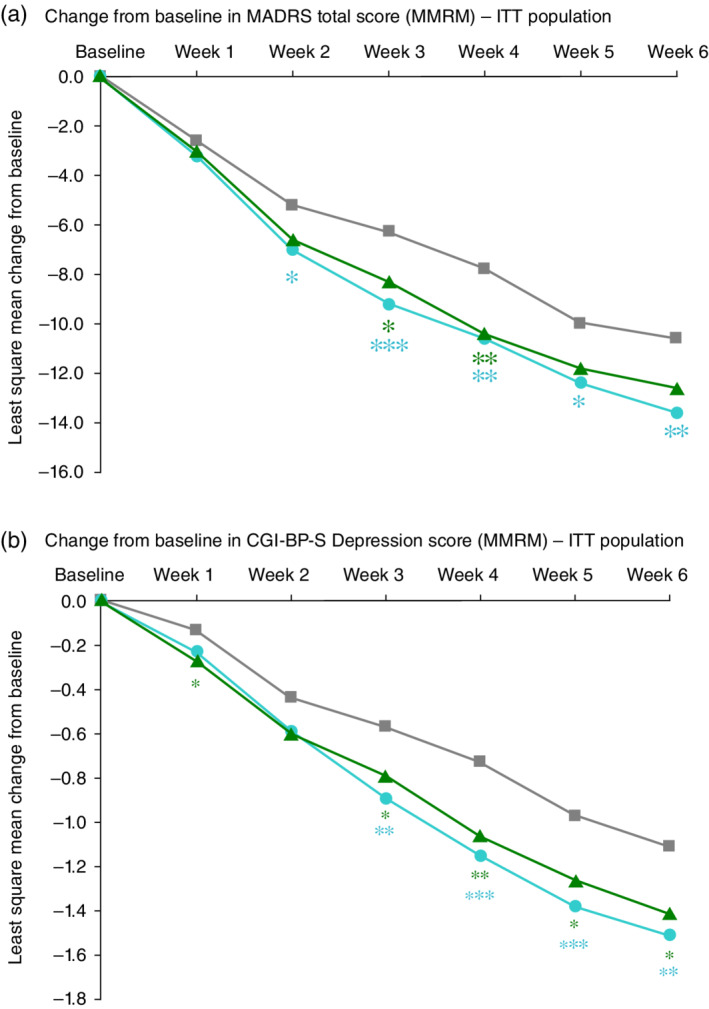
(a) Change from baseline in Montgomery–Åsberg Depression Rating Scale (MADRS) total score (mixed‐effects model for repeated measures [MMRM]) – intent‐to‐treat (ITT) population. *P < 0.05, **P < 0.01, ***P < 0.001 (vs placebo). Adjustments for multiple comparisons by Hochberg (only at Week 6) 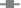, Placebo (*n* = 171); 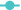, Lurasidone 20–60 mg/day (*n* = 182); 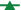, Lurasidone 80–120 mg/day (*n* = 169). (b) Change from baseline in Clinical Global Impression: Bipolar Version – Severity of Illness (CGI‐BP‐S) Depression score (MMRM) – ITT population. *P < 0.05, **P < 0.01, ***P < 0.001 (vs placebo). No adjustments for multiple comparisons 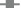, Placebo (*n* = 171); 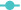, Lurasidone 20–60 mg/day (*n* = 182); 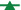, Lurasidone 80–120 mg/day (*n* = 169).

On the primary end‐point, the lurasidone 20–60‐mg/day group, but not the lurasidone 80–120‐mg/day group, was significantly superior to placebo. The LS mean change (±SE) in the MADRS total score from baseline to Week 6 was −13.6 ± 0.69 (adjusted *P* = 0.007; effect size = 0.33 vs placebo) for the lurasidone 20–60‐mg/day group, −12.6 ± 0.73 (adjusted *P* = 0.057; effect size = 0.22 vs placebo) for the lurasidone 80–120‐mg/day group, and −10.6 ± 0.72 for the placebo group (Fig. 2a). In the 80–120‐mg/day group, effect sizes for modal daily lurasidone doses of 80 mg, 100 mg, and 120 mg were 0.34, 0.27, and 0.06, respectively, based on a *post‐hoc* analysis. In the lurasidone 20–60‐mg/day group, superiority compared with placebo on the MADRS was observed from Week 2 through to Week 6 (Fig. 2a). Both lurasidone treatment groups showed significantly greater improvement from baseline to Week 6 compared with placebo in core depressive symptoms (MADRS‐6 subscale score; Table S1).

The proportion of patients with a response on the MADRS total score was significantly greater (*P* = 0.003) in the lurasidone 20–60‐mg/day group (84 patients, 46.2% [NNT = 7]) but not in the lurasidone 80–120‐mg/day group (65 patients, 38.5%; *P* = 0.145) compared with the placebo group (31.0%). Remission was achieved by 31.9% of those in the lurasidone 20–60‐mg/day group (*P* = 0.064 vs placebo), 29.0% of those in the lurasidone 80–120‐mg/day group (*P* = 0.172 vs placebo), and 22.8% of those in the placebo group. Results for the time‐to‐response analyses based on a Kaplan–Meier estimate showed a probability of response by Week 6 (up to Day 42) of 53.1% for the lurasidone 20–60‐mg/day group (*P* = 0.02 vs placebo), 51.6% for the lurasidone 80–120‐mg/day group (*P* = 0.054 vs placebo), and 39.3% for the placebo group. The Cox proportional hazard ratio of MADRS response was significantly higher for the lurasidone 20–60‐mg/day group (1.5; 95% confidence interval [CI], 1.1–2.1; *P* = 0.010) and for the lurasidone 80–120‐mg/day group (1.5; 95%CI, 1.0–2.0; *P* = 0.025) compared with the placebo group.

Improvement in the CGI‐BP‐S Depression score from baseline to Week 6 was significantly different for both lurasidone treatment groups compared with placebo (MMRM). The LS mean change (±SE) from baseline to Week 6 was −1.51 ± 0.09 (*P* = 0.002; effect size = 0.35 vs placebo) for the lurasidone 20–60‐mg/day group, −1.41 ± 0.09 (*P* = 0.019; effect size = 0.27 vs placebo) for the lurasidone 80–120‐mg/day group, and −1.11 ± 0.09 for the placebo group (Fig. 2b). For both dosages of lurasidone, significantly greater reduction compared with placebo on the CGI‐BP‐S Depression score was observed starting at Week 3 and this was maintained at all subsequent study visits.

Treatment with lurasidone 20–60 mg/day was associated with significant improvement from baseline to Week 6 compared with placebo in functioning, as measured by the patient‐rated SDS, and in anxiety symptoms, as measured by the HAM‐A (Table 2). No significant differences were evident comparing the lurasidone 80–120‐mg/day group to placebo on the HAM‐A or SDS. The YMRS total scores were slightly decreased from baseline at Week 6 in all groups despite the low baseline score (Table 2).

**Table 2:** Secondary efficacy and safety measures: Least square mean change (SE) from baseline to Week 6 (intent‐to‐treat population)

	Lurasidone 20–60 mg/day (*n* = 182)			Lurasidone 80–120 mg/day (*n* = 169)			Placebo (*n* = 171)		
Parameter	*n*	Baseline mean (SD)	Mean change (SE)	*n*	Baseline mean (SD)	Mean change (SE)	*n*	Baseline mean (SD)	Mean change (SE)
Efficacy measure (LOCF, ANCOVA)									
SDS	147	19.4 (5.3)	−7.6 (0.6)*	124	19.8 (5.6)	−6.8 (0.7)	128	20.3 (5.0)	−5.7 (0.7)
HAM‐A	179	17.6 (7.3)	−7.4 (0.5)*	167	16.7 (6.2)	−6.4 (0.5)	164	17.2 (6.6)	−5.7 (0.5)
YMRS	182	2.92 (2.49)	−0.64 (0.22)	169	2.67 (2.29)	−0.87 (0.23)*	171	2.62 (2.23)	−0.22 (0.23)
Safety measure (MMRM)									
DIEPSS	182	0.41 (1.01)	−0.01 (0.08)	169	0.39 (0.83)	0.39 (0.09)**	171	0.36 (0.93)	−0.02 (0.09)

* *P* < 0.05; **P < 0.01, comparisons to placebo group.

ANCOVA, analyses of covariance; DIEPSS, Drug‐Induced Extrapyramidal Symptoms Scale total score (excluding severity); HAM‐A, Hamilton Anxiety Rating Scale total score; LOCF, last observation carried forward; MMRM, mixed‐effects model for repeated measures; SDS, Sheehan Disability Scale total score; YMRS: Young Mania Rating Scale total score.

### Safety

Overall, treatment with lurasidone 20–60 mg/day and 80–120 mg/day was safe and well tolerated. TEAEs were reported by 53.3% of patients in the lurasidone 20–60‐mg/day group, 59.2% of patients in the lurasidone 80–120‐mg/day group, and 45.9% of patients in the placebo group. The majority of TEAEs reported were classified as either mild or moderate in severity. Severe TEAEs were reported for three patients (1.6%) in the lurasidone 20–60‐mg/day group, nine patients (5.3%) in the lurasidone 80–120‐mg/day group, and two patients (1.2%) in the placebo group.

Serious adverse events during treatment were reported for two patients (1.1%) in the lurasidone 20–60‐mg/day group, four patients (2.4%) in the lurasidone 80–120‐mg/day group, and five patients (2.9%) in the placebo group. Treatment discontinuation due to a TEAEs was reported for six patients (3.3%) in the lurasidone 20–60‐mg/day group, 16 patients (9.5%) in the lurasidone 80–120‐mg/day group, and seven patients (4.1%) in the placebo group. There were no treatment‐emergent deaths reported during the 6‐week treatment phase of the study.

The most common TEAEs were akathisia, nausea, and somnolence (Table 3). These TEAEs (akathisia, nausea, somnolence) were more frequent in the lurasidone 80–120‐mg/day group (23.7%, 11.8%, 6.5%, respectively) compared to the 20–60‐mg/day group (13.0%, 6.5%, 3.8%, respectively). Other common TEAEs in the lurasidone groups with a rate greater than in the placebo group were parkinsonism and nasopharyngitis (Table 3). All but two cases of akathisia were reported as mild or moderate in severity. Nausea was the only TEAEs leading to discontinuation in more than 2% of patients in a group (2.4% in the lurasidone 80–120‐mg/day group).

**Table 3:** Treatment‐emergent adverse events (safety population; *n* [%])

	Lurasidone	Lurasidone	Placebo
	20–60 mg/day	80–120 mg/day	
	(*n* = 184)	(*n* = 169)	(*n* = 172)
At least one event	98 (53.3%)	100 (59.2%)	79 (45.9%)
Event (incidence ≥ 5%)			
Akathisia	24 (13.0%)	40 (23.7%)	11 (6.4%)
Nausea	12 (6.5%)	20 (11.8%)	10 (5.8%)
Somnolence	7 (3.8%)	11 (6.5%)	7 (4.1%)
Nasopharyngitis	10 (5.4%)	6 (3.6%)	8 (4.7%)
Headache	5 (2.7%)	9 (5.3%)	15 (8.7%)
Parkinsonism	4 (2.2%)	10 (5.9%)	4 (2.3%)

Treatment‐emergent mania occurred in seven patients (3.8%) in the lurasidone 20–60‐mg/day group, three patients (1.8%) in the lurasidone 80–120‐mg/day group, and four patients (2.3%) in the placebo group. Odds ratios for developing treatment‐emergent mania compared with placebo were 2.3 (95%CI, 0.4–12.4; *P* = 0.334) for the lurasidone 20–60‐mg/day group and 1.1 (95%CI, 0.1–8.1; *P* = 0.939) for the lurasidone 80–120‐mg/day group. Based on the C‐SSRS, there was no notable difference in the proportion of patients with at least one post‐baseline instance of suicidality (defined as suicidal ideation or behavior) comparing the lurasidone 20–60‐mg/day group (27 patients, 14.8%) and the lurasidone 80–120‐mg/day group (24 patients, 14.2%) with the placebo group (27 patients, 15.8%). Emergence of suicidal behavior after baseline was reported for one patient (0.5%) in the lurasidone 20–60‐mg/day group, one patient (0.6%) in the lurasidone 80–120‐mg/day group, and no patients in the placebo group.

TEAEs related to extrapyramidal symptoms were reported for eight patients (4.3%) in the lurasidone 20–60‐mg/day group, 18 patients (10.7%) in the lurasidone 80–120‐mg/day group, and 11 patients (6.4%) in the placebo group. There were no significant differences between the DIEPSS total scores for the lurasidone 20–60‐mg/day group and the placebo group at any of the study visits. There was a significant difference between the lurasidone 80–120‐mg/day group and the placebo group for change from baseline to Week 6 in the DIEPSS total score. However, mean change (±SE) in total score from baseline to Week 6 was very small in all groups (Table 2). One or more concomitant antiparkinson medications was recorded for 6.0% of patients in the lurasidone 20–60‐mg/day group, 20.7% of patients in the lurasidone 80–120‐mg/day group, and 3.5% of patients in the placebo group.

Metabolic‐related TEAEs were reported for five patients (2.7%) in the lurasidone 20–60‐mg/day group, one patient (0.6%) in the lurasidone 80–120‐mg/day group, and four patients (2.3%) in the placebo group. There were no clinically relevant changes from baseline to Week 6 (LOCF) in metabolic parameters in patients receiving lurasidone (either dose) compared with patients receiving placebo (Table 4).

**Table 4:** Baseline and mean change to Week 6 (LOCF) in laboratory parameters (safety population)

	Lurasidone 20–60 mg/day (*n* = 184)				Lurasidone 80–120 mg/day (*n* = 169)				Placebo (*n* = 172)			
Parameter	*n*	Baseline mean (SD)	Mean change (SD)	Median change	*n*	Baseline mean (SD)	Mean change (SD*)*	Median change	*n*	Baseline mean (SD)	Mean change (SD)	Median change
Total cholesterol (mg/dl)^†^	164	205.2 (44.3)	−5.6 (28.9)	0.0	151	199.8 (40.9)	−4.2 (35.1)	−7.0	158	201.9 (44.4)	−4.6 (28.2)	−4.5
LDL cholesterol (mg/dL)^†^	160	122.0 (38.8)	−4.7 (26.6)	−4.0	145	118.4 (38.1)	−3.1 (28)	−4.0	152	118.2 (38.0)	−3.5 (24.3)	−2.5
Triglycerides (mg/dL)^†^	164	142.8 (93.3)	−7.9 (74.5)	−6.0	151	138.4 (86.3)	8.8 (128.1)	−2.0	158	131.1 (95.3)	−0.8 (68.8)	0.5
Glucose (mg/dL)^†^	163	96.2 (13.8)	−1.1 (13.9)	−2.0	151	96.7 (14.3)	0.3* (12.7)	1.0	159	97.1 (14.7)	−2.9 (13.1)	−2.0
HbA1c (%)	174	5.32 (0.41)	0.01 (0.21)	0.00	164	5.30 (0.43)	0.02* (0.25)	0.00	164	5.29 (0.39)	−0.02 (0.22)	0.00
Insulin (mU/L)^†^	173	13.1 (13.1)	0.4 (18.6)	−0.1	157	12.9 (10.4)	2.8 (13.2)	−0.2	163	15.2 (17.5)	−1.5 (19.1)	−0.3
Prolactin (ng/mL), overall	176	9.9 (11.6)	3.5** (17.0)	1.8	167	14.2 (28.8)	4.3** (18.9)	2.8	165	13.2 (19.7)	−1.5 (20.3)	0.0
Prolactin (ng/mL), male	83	7.3 (6.3)	1.9** (7.8)	1.2	79	8.8 (10.7)	2.4** (13.3)	2.7	75	10.5 (11.6)	−3.0 (10.3)	−0.4
Prolactin (ng/mL), female	93	12.3 (14.5)	5.0** (22.1)	2.6	88	19.1 (37.9)	6.1** (22.8)	3.5	90	15.5 (24.3)	−0.4 (25.9)	0.5
Weight (kg)	179	72.0 (13.7)	0.16** (1.85)	0.2	166	73.9 (16.8)	0.0 (2.03)	0.0	166	71.3 (14.3)	−0.29 (1.49)	−0.1
Body mass index (kg/m^2^)	179	25.6 (4.1)	0.07** (0.66)	0.07	166	26.1 (5.0)	0.0 (0.71)	0.00	166	25.3 (4.3)	−0.1 (0.54)	−0.03

^†^ Fasting condition.

* *P* < 0.05; **P < 0.01, comparisons to placebo group.

LOCF, last observation carried forward.

Prolactin concentrations from baseline to Week 6 (LOCF) were increased in the lurasidone 20–60‐mg/day group and in the lurasidone 80–120‐mg/day group relative to the placebo group (*P* < 0.01 for both groups; Table 4). Markedly abnormal post‐baseline change in prolactin (≥5‐fold of upper limit of normal) occurred in 1.1% (two patients) in the lurasidone 20–60‐mg/day group, 0.6% (one patient) in the lurasidone 80–120‐mg/day group, and no patients in the placebo group. There was no evidence of toxicity as measured by clinical laboratory, hematological, and urinalysis parameters of lurasidone‐treated patients compared with placebo‐treated patients.

Analysis of vital signs and physical findings did not reveal any clinically relevant effect of lurasidone treatment in the full sample. There was no effect of lurasidone when compared with placebo on pulse rate, blood pressure (systolic or diastolic), or body temperature. There were no clinically relevant effects on weight or body mass index (Table 4). The proportions of patients with ≥7% weight increase/decrease were 1.1%/1.1%, 1.2%/0.6%, and 0%/0% in the lurasidone 20–60‐mg/day group, the lurasidone 80–120‐mg/day group, and the placebo group, respectively. There were no clinically relevant changes from baseline to Week 6 in ECG parameters in any of the treatment groups.

## Discussion

This 6‐week, multicenter, international study found that monotherapy with lurasidone (20–60 mg/day) for bipolar I depression was significantly superior to placebo in reducing depressive symptoms as measured by the MADRS total score (primary end‐point), with improvement evident as early as Week 2. Significantly greater improvement for the lurasidone 20–60‐mg/day group, compared to placebo, was also evident on the following secondary measures at Week 6: responder rates, Kaplan–Meier estimate of the probability of response, improvement in global illness severity (CGI‐BP‐S Depression score), reduction in anxiety symptoms (HAM‐A), and improvement in patient‐rated functioning (on the SDS).

A meta‐analysis of placebo‐controlled, monotherapy trials of mood‐stabilizing anticonvulsants, second‐generation antipsychotics, and lithium for bipolar depression reported an overall NNT of 8.2 for a comparison of responder rates versus placebo.^34^ The NNT value of lurasidone 20–60 mg/day was 7 and in the same range as reported in the meta‐analysis, suggesting clinical significance. The efficacy profile of lurasidone 20–60 mg/day is consistent with the previous study of lurasidone as monotherapy for bipolar I depression conducted mainly in the USA and Europe,^20^ suggesting that lurasidone has broad efficacy across various ethnic populations.

In the previous monotherapy study,^20^ lurasidone 80–120 mg/day significantly reduced depressive symptoms compared to placebo on the primary end‐point. However, in the current study the higher (80–120 mg/day) dose range of lurasidone did not demonstrate significant improvement on the MADRS (adjusted *P* = 0.057).

In the current study, the modal daily dose used by 30.2% of the patients in the lurasidone 80–120‐mg/day dosage group was the maximum dose of 120 mg, which was almost two‐fold higher than the rate reported in the previous monotherapy study (16.1%).^20^ This may be partly attributable to dose escalation criteria added in the current protocol that encouraged continued dose increases until depression severity was reduced (CGI‐BP‐S Depression score ≤ 4). Patients treated with higher doses may achieve higher plasma concentrations of lurasidone, which in turn have been correlated with notably higher dopamine D2 receptor occupancy levels.^35^ It has been suggested that high levels of D2 receptor occupancy may be associated with dysphoria.^36^ In the current study, the lower effect size observed on the modal daily dose of 120 mg of lurasidone may be attributable to this D2 receptor occupancy effect, and this may be associated with reduced efficacy in the high‐dose group.

The safety profile for lurasidone in the current study was comparable to that reported in previous trials. The most common TEAEs was akathisia, which occurred with a higher frequency in the current study (vs the previous monotherapy study^20^) both in the 20–60‐mg/day group (13.0% vs 7.9%), and in the 80–120‐mg/day group (23.7% vs 10.8%). It is notable that the rate of akathisia reported in the placebo group was also higher in the current study (vs the previous monotherapy study: 6.4% vs 2.4%), resulting in similar placebo‐adjusted rates for both studies. Consistent with this, in the current study, no patients in the lurasidone 20–60‐mg/day group reported severe akathisia, and rates of treatment discontinuation due to akathisia were low and similar to placebo (0.6% vs 0.5%). Among patients in the lurasidone 80–120‐mg/day group, two reported severe akathisia and discontinuation due to akathisia was 1.8%. Treatment discontinuation due to any TEAEs was also low in the lurasidone 20–60‐mg/day group and comparable to placebo (3.3% vs 4.1%), and was somewhat higher in the 80–120‐mg/day group (9.5%).

In comparing lurasidone dose groups to placebo, there were no differences in rates of treatment‐emergent mania. This is notable given the risk of treatment‐emergent mania that has been reported when using antidepressants in the treatment of bipolar depression.^37^ YMRS scores were low at baseline and remained unchanged after 6 weeks of treatment with both doses of lurasidone. Previous studies indicate that long‐term treatment with lurasidone in bipolar depression is associated with a low risk of triggering a manic switch; and in one double‐blind maintenance trial in bipolar depression, lurasidone was associated with a 43% reduction in the risk of developing mania versus placebo (both treatment groups utilized adjunctive lithium or valproate).^38^, ^39^ Many antipsychotics have demonstrated efficacy in treatment of acute manic and mixed manic episodes; however, randomized trials of lurasidone have not been reported.^40^

The results of the current study found lurasidone to have minimal effects on weight and metabolic parameters. Multiple studies of lurasidone in patients with both bipolar disorder and schizophrenia have reported similar findings.^39^, ^41^, ^42^ As bipolar disorder is associated with high rates of metabolic syndrome and cardiovascular mortality,^43^, ^44^ the favorable metabolic profile of lurasidone is an important safety consideration, especially given the frequent need for long‐term therapy in bipolar disorder patients.

Lurasidone treatment was associated with a small increase in prolactin that was not considered to be clinically relevant, as was found in the previous monotherapy study.^20^ No new safety concerns or risks were apparent for lurasidone in the present study, suggesting that lurasidone is safe and well tolerated across various ethnic populations.

Olanzapine and quetiapine XR other than lurasiodne are the only approved medications for bipolar depression in Japan. With these medications, two randomized, double‐blind placebo‐controlled studies that enrolled Japanese patients have been reported. In a 6‐week study of olanzapine in bipolar I patients, 30% of the treatment sample were Japanese.^45^ In an 8‐week trial of quetiapine XR, all enrolled patients were Japanese (*n* = 431), with 29% of these receiving a bipolar I depression diagnosis.^46^ The current 6‐week flexible‐dose study of lurasidone for the treatment of bipolar I patients included 34% Japanese patients. All three antipsychotics showed significant antidepressant effect in MADRS total score compared to placebo. Interestingly, individual MADRS items showing greater improvement may differ depending on the antipsychotics; that is, efficacy was apparent for sadness and inner tension with lurasidone (see Table S1), while efficacy regarding reduced sleep and reduced appetite was evident with olanzapine.^45^

The safety and tolerability profiles also differ for lurasidone compared with olanzapine and quetiapine XR. In terms of adverse events, lurasidone was associated with akathisia and nausea, olanzapine with somnolence and weight increase,^45^ and quetiapine XR with somnolence and thirst.^46^ In the current study (similar to results from previous studies^20^, ^21^, ^38^), lurasidone was not associated with increases in weight or lipid parameters. The lack of weight gain may be explained by the receptor binding profile of lurasidone, which has no appreciable affinity for histamine H_1_ (IC_50_, >1000 nM) or 5‐HT_2C_ (Ki, 415 nM). Inhibitory activity at these receptors has been shown to be associated with weight gain.^47^, ^48^ Both olanzapine and quetiapine have higher affinity for histamine H_1_ and 5‐HT_2C_ receptors, which have been shown to be associated with glucose intolerance and increased insulin resistance,^47^, ^49^, ^50^, ^51^ and both have a known characteristic to increase weight and elevate lipids.^52^, ^53^

Although detailed comparisons must be made cautiously given cross‐study differences in trial design and study populations, lurasidone should be a valuable option for the treatment of bipolar depression in Japan with a different efficacy/safety profile.

A few limitations of this study should be noted. Patients with serious psychiatric or medical comorbidities were excluded, as were patients with bipolar II disorder, and therefore the generalizability of the current findings to patients with those characteristics is unknown. The extent to which lurasidone is safe and effective over longer periods of time in Asian populations (including Japanese patients) needs further investigation.

In conclusion, this study demonstrated that monotherapy with lurasidone 20–60 mg/day was efficacious, relative to placebo, in the treatment of depressive symptoms in patients with bipolar I disorder. Improvement was also observed in global severity of illness, concomitant anxiety, and in functioning. Lurasidone was generally well‐tolerated, especially at the 20–60‐mg/day dose range, with low rates of discontinuation due to adverse events (3.3%). The higher dose of 80–120 mg/day was less effective and had higher rates of adverse events. Lurasidone had minimal effects on weight and metabolic parameters, and was associated with a low risk of switching to mania. These results are consistent with findings from a previously reported monotherapy study of lurasidone in bipolar I depression,^20^ suggesting that lurasidone 20–60 mg/day is effective and safe to treat bipolar I depression in diverse ethnic populations, including Japanese.

## Disclosure statement

Tadafumi Kato reports personal fees from Kyowa Hakko Kirin, Eli Lilly, Otsuka, GlaxoSmithKline, Taisho Toyama, Sumitomo Dainippon, Meiji Seika Pfizer, Mochida, Shionogi, Janssen, Janssen Asia Pacific, Yoshitomiyakuhin, Astellas, Wako Pure Chemical Industries, Wiley Publishing Japan, Nippon Boehringer Ingelheim Kanae Foundation for the Promotion of Medical Science, MSD, Kyowa, and Takeda, and also reports a research grant from Takeda. Teruhiko Higuchi reports personal fees from Meiji Seika Pharma, MSD, Allergan, Eisai, Pfizer, Janssen, Lundbeck, Shionogi, Yoshitomi, Kyowa Hakko Kirin, Mochida, Otsuka, Sumitomo Dainippon, Mitsubishi Tanabe, Eli Lilly, and Takeda. Jun Ishigooka reports grants from Sumitomo Dainippon during the conduct of the study, personal fees from Meiji Seika Pharma, MSD, Astellas, Novartis, Pfizer, Otsuka, Eli Lilly, Takeda, and Eisai. Eduard Vieta reports personal fees from Abbott, Angelini, Janssen, Lundbeck, Sage, and Sanofi, and reports grants from Sumitomo Dainippon, Ferrer, and Janssen outside the submitted work. Mari Miyajima, Kei Watabe, and Takahiro Masuda are full‐time employees of Sumitomo Dainippon Pharma Co., Ltd. Tomohiro Fujimori used to be a full‐time employee of Sumitomo Dainippon Pharma Co., Ltd. during the conduct of the study, and is a full‐time employee of Sunovion Pharmaceuticals Inc.

## Author contributions

T.K. contributed to the acquisition and interpretation of data. J.I. and T.H. contributed to the conception and design of the study, acquisition and interpretation of data. K.W. contributed to the conception and design of the study and analysis and interpretation of data. M.M. and T.M. contributed to analysis and interpretation of data. T.F. contributed to the acquisition of data. E.V. contributed to the interpretation of data. All authors contributed to and approved the final draft prior to submission.

## Supporting information


**Supplemental Table S1.** Individual Montgomery–Åsberg Depression Rating Scale (MADRS) Item Score and MADRS‐6 subscale: Change from baseline to Week 6 (intent‐to‐treat population, mixed effects model for repeated measures).

## References

[1] Merikangas KR , Jin R , He JP *et al* Prevalence and correlates of bipolar spectrum disorder in the World Mental Health Survey initiative. Arch. Gen. Psychiatry 2011; 68: 241–251. 21383262 10.1001/archgenpsychiatry.2011.12 PMC3486639

[2] Nishi D , Ishikawa H , Kawakami N . Prevalence of mental disorders and mental health service use in Japan. Psychiatry Clin. Neurosci. 2019; 73: 458–465. 31141260 10.1111/pcn.12894

[3] Vos T , Flaxman AD , Naghavi M *et al* Years lived with disability (YLDs) for 1160 sequelae of 289 diseases and injuries 1990–2010: A systematic analysis for the Global Burden of Disease Study 2010. Lancet 2012; 380: 2163–2169. 23245607 10.1016/S0140-6736(12)61729-2 PMC6350784

[4] Fajutrao L , Locklear J , Priaulx J , Heyes A . A systematic review of the evidence of the burden of bipolar disorder in Europe. Clin. Pract. Epidemiol. Mental Health 2009; 5: 3. 10.1186/1745-0179-5-3 PMC2646705 19166608

[5] Gardner HH , Kleinman NL , Brook RA , Rajagopalan K , Brizee TJ , Smeeding JE . The economic impact of bipolar disorder in an employed population from an employer perspective. J. Clin. Psychiatry 2006; 67: 1209–1218. 16965198 10.4088/jcp.v67n0806

[6] Dean BB , Gerner D , Gerner RH . A systematic review evaluating health‐related quality of life, work impairment, and healthcare costs and utilization in bipolar disorder. Curr. Med. Res. Opin. 2004; 20: 139–154. 15006007 10.1185/030079903125002801

[7] Crump C , Sundquist K , Winkleby MA , Sundquist J . Comorbidities and mortality in bipolar disorder: A Swedish national cohort study. JAMA Psychiatry 2013; 70: 931–939. 23863861 10.1001/jamapsychiatry.2013.1394

[8] Correll CU , Solmi M , Veronese N *et al* Prevalence, incidence and mortality from cardiovascular disease in patients with pooled and specific severe mental illness: A large‐scale meta‐analysis of 3,211,768 patients and 113,383,368 controls. World Psychiatry 2017; 16: 163–180. 28498599 10.1002/wps.20420 PMC5428179

[9] Vancampfort D , Correll CU , Galling B *et al* Diabetes mellitus in people with schizophrenia, bipolar disorder and major depressive disorder: A systematic review and large scale meta‐analysis. World Psychiatry 2016; 15: 166–174. 27265707 10.1002/wps.20309 PMC4911762

[10] Walker ER , McGee RE , Druss BG . Mortality in mental disorders and global disease burden implications: A systematic review and meta‐analysis. JAMA Psychiatry 2015; 72: 334–341. 25671328 10.1001/jamapsychiatry.2014.2502 PMC4461039

[11] Dilsaver SC . An estimate of the minimum economic burden of bipolar I and II disorders in the United States. J. Affect. Disord. 2011; 129: 79–83. 20888048 10.1016/j.jad.2010.08.030

[12] Watanabe K , Harada E , Inoue T , Tanji Y , Kikuchi T . Perceptions and impact of bipolar disorder in Japan: Results of an internet survey. Neuropsychiatr. Dis. Treat. 2016; 12: 2981–2987. 27920534 10.2147/NDT.S113602 PMC5123658

[13] Calabrese JR , Hirschfeld RM , Frye MA , Reed ML . Impact of depressive symptoms compared with manic symptoms in bipolar disorder: Results of a US community based sample. J. Clin. Psychiatry 2004; 65: 1499–1504. 15554762 10.4088/jcp.v65n1109

[14] Judd LL , Akiskal HS , Schettler PJ *et al* The long term natural history of the weekly symptomatic status of bipolar I disorder. Arch. Gen. Psychiatry 2002; 59: 530–537. 12044195 10.1001/archpsyc.59.6.530

[15] Vieta E , Berk M , Schulze TG *et al* Bipolar disorders. Nat. Rev. Dis. Primers. 2018; 4: 18008. 29516993 10.1038/nrdp.2018.8

[16] Kato T . Current understanding of bipolar disorder: Toward integration of biological basis and treatment strategies. Psychiatry Clin. Neurosci. 2019; 73: 526–540. 31021488 10.1111/pcn.12852

[17] Ishibashi T , Horisawa T , Tokuda K *et al* Pharmacological profile of lurasidone, a novel antipsychotic agent with potent 5‐hydroxytryptamine 7 (5‐HT7) and 5‐HT1A receptor activity. J. Pharmacol. Exp. Ther. 2010; 334: 171–181. 20404009 10.1124/jpet.110.167346

[18] Hedlund PB . The 5‐HT7 receptor and disorders of the nervous system: An overview. Psychopharmacology 2009; 206: 345–354. 19649616 10.1007/s00213-009-1626-0 PMC2841472

[19] Cates LN , Roberts AJ , Huitron‐Resendiz S , Hedlund PB . Effects of lurasidone in behavioral models of depression: Role of the 5‐HT_7_ receptor subtype. Neuropharmacology 2013; 70: 211–217. 23416039 10.1016/j.neuropharm.2013.01.023

[20] Loebel A , Cucchiaro J , Silva R *et al* Lurasidone monotherapy in the treatment of bipolar I depression: A randomized, double‐blind, placebo‐controlled study. Am. J. Psychiatry 2014; 171: 160–168. 24170180 10.1176/appi.ajp.2013.13070984

[21] Loebel A , Cucchiaro J , Silva R *et al* Lurasidone as adjunctive therapy with lithium or valproate for the treatment of bipolar I depression: A randomized, double‐blind, placebo‐controlled study. Am. J. Psychiatry 2014; 171: 169–177. 24170221 10.1176/appi.ajp.2013.13070985

[22] Fountoulakis KN . The International College of Neuro‐Psychopharmacology (CINP) treatment guidelines for bipolar disorder in adults (CINP‐BD‐2017), Part 3: The clinical guidelines. Int. J. Neuropsychopharmacol. 2017; 20: 180–195. 27941079 10.1093/ijnp/pyw109 PMC5408976

[23] Malhi GS . Royal Australian and New Zealand College of Psychiatrists clinical practice guidelines for mood disorders. Aust. N. Z. J. Psychiatry 2015; 49: 1087–1206. 26643054 10.1177/0004867415617657

[24] Yatham LN . Canadian Network for Mood and Anxiety Treatments (CANMAT) and International Society for Bipolar Disorders (ISBD) 2018 guidelines for the management of patients with bipolar disorder. Bipolar Disord. 2018; 20: 97–170. 29536616 10.1111/bdi.12609 PMC5947163

[25] Sheehan DV , Lecrubier Y , Sheehan KH *et al* The MINI‐International Neuropsychiatric Interview (MINI): The development and validation of a structured diagnostic psychiatric interview for DSMIV and ICD‐10. J. Clin. Psychiatry 1998; 59: 22–33. 9881538

[26] Montgomery SA , Åsberg M . A new depression scale designed to be sensitive to change. Br. J. Psychiatry 1979; 134: 382–389. 444788 10.1192/bjp.134.4.382

[27] Young RC , Biggs JT , Ziegler VE , Meyer DA . A rating scale for mania: Reliability, validity and sensitivity. Br. J. Psychiatry 1978; 133: 429–435. 728692 10.1192/bjp.133.5.429

[28] Chappell P , Feltner DE , Makumi C , Stewart M . Initial validity and reliability data on the Columbia‐Suicide Severity Rating Scale. Am. J. Psychiatry 2012; 169: 662–663. 10.1176/appi.ajp.2012.12010123 22684597

[29] Spearing MK , Post RM , Leverich GS , Brandt D , Nolen W . Modification of the Clinical Global Impressions (CGI) Scale for use in bipolar illness (BP): The CGI‐BP. Psychiatry Res. 1997; 73: 159–171. 9481807 10.1016/s0165-1781(97)00123-6

[30] Sheehan DV . The Anxiety Disease. Bantam Books, New York, NY, 1983.

[31] Hamilton M . The assessment of anxiety states by rating. Br. J. Med. Psychol. 1959; 32: 50–55. 13638508 10.1111/j.2044-8341.1959.tb00467.x

[32] Bech P , Tanghøj P , Andersen HF , Overø K . Citalopram dose‐response revisited using an alternative psychometric approach to evaluate clinical effects of four fixed citalopram doses compared to placebo in patients with major depression. Psychopharmacology 2002; 163: 20–25. 12185396 10.1007/s00213-002-1147-6

[33] Inada T , Beasley CM Jr , Tanaka Y , Walker DJ . Extrapyramidal symptom profiles assessed with the Drug‐Induced Extrapyramidal Symptom Scale: Comparison with Western scales in the clinical double‐blind studies of schizophrenic patients treated with either olanzapine or haloperidol. Int. Clin. Psychopharmacol. 2003; 18: 39–48. 12490774 10.1097/00004850-200301000-00007

[34] Selle V , Schalkwijk S , Vázquez GH , Baldessarini RJ . Treatments for acute bipolar depression: Meta‐analyses of placebo‐controlled, monotherapy trials of anticonvulsants, lithium and antipsychotics. Pharmacopsychiatry 2014; 47: 43–52. 24549862 10.1055/s-0033-1363258

[35] Potkin SG , Keator DB , Kesler‐West ML *et al* D2 receptor occupancy following lurasidone treatment in patients with schizophrenia or schizoaffective disorder. CNS Spectr. 2014; 19: 176–181. 24073841 10.1017/S109285291300059X

[36] de Haan L , Lavalaye J , van Bruggen M *et al* Subjective experience and dopamine D2 receptor occupancy in patients treated with antipsychotics: Clinical implications. Can. J. Psychiatry 2004; 49: 290–296. 15198464 10.1177/070674370404900503

[37] Viktorin A , Lichtenstein P , Thase ME *et al* The risk of switch to mania in patients with bipolar disorder during treatment with an antidepressant alone and in combination with a mood stabilizer. Am. J. Psychiatry 2014; 171: 1067–1073. 24935197 10.1176/appi.ajp.2014.13111501

[38] Ketter TA , Sarma K , Silva R , Kroger H , Cucchiaro J , Loebel A . Lurasidone in the long‐term treatment of patients with bipolar disorder: A 24‐week open‐label extension study. Depress. Anxiety 2016; 33: 424–434. 26918425 10.1002/da.22479 PMC5069590

[39] Calabrese JR , Pikalov A , Streicher C , Cucchiaro J , Mao Y , Loebel A . Lurasidone in combination with lithium or valproate for the maintenance treatment of bipolar I disorder. Eur. Neuropsychopharmacol. 2017; 27: 865–876. 28689688 10.1016/j.euroneuro.2017.06.013

[40] Ketter TA , Miller S , Dell'Osso B , Calabrese JR , Frye MA , Citrome L . Balancing benefits and harms of treatments for acute bipolar depression. J. Affect. Disord. 2014; 169: S24–S33. 25533911 10.1016/S0165-0327(14)70006-0

[41] Barton BB , Segger F , Fischer K , Obermeier M , Musil R . Update on weight‐gain caused by antipsychotics: A systematic review and meta‐analysis. Expert Opin. Drug Saf. 2020; 19: 295–314. 31952459 10.1080/14740338.2020.1713091

[42] Huhn M , Nikolakopoulou A , Schneider‐Thoma J *et al* Comparative efficacy and tolerability of 32 oral antipsychotics for the acute treatment of adults with multi‐episode schizophrenia: A systematic review and network meta‐analysis. Lancet 2019; 394: 939–951. 31303314 10.1016/S0140-6736(19)31135-3 PMC6891890

[43] de Jong M , Belleflamme J , Dale C *et al* Metabolic syndrome in Dutch patients with bipolar disorder: A cross‐sectional study. Prim. Care Companion CNS Disord. 2018; 20: 18m02366. 10.4088/PCC.18m02366 30549480

[44] Vancampfort D , Vansteelandt K , Correll CU *et al* Metabolic syndrome and metabolic abnormalities in bipolar disorder: A meta‐analysis of prevalence rates and moderators. Am. J. Psychiatry 2013; 170: 265–274. 23361837 10.1176/appi.ajp.2012.12050620

[45] Tohen M , McDonnell DP , Case M *et al* Randomised, double‐blind, placebo‐controlled study of olanzapine in patients with bipolar I depression. Br. J. Psychiatry 2012; 201: 376–382. 22918966 10.1192/bjp.bp.112.108357

[46] Murasaki M , Koyama T , Kanba S *et al* Multi‐center, randomized, double‐blind, placebo‐controlled study of quetiapine extended‐release formulation in Japanese patients with bipolar depression. Psychopharmacology 2018; 235: 2859–2869. 30069587 10.1007/s00213-018-4977-6 PMC6182597

[47] Reynolds GP , Kirk SL . Metabolic side effects of antipsychotic drug treatment–pharmacological mechanisms. Pharmacol. Ther. 2010; 125: 169–179. 19931306 10.1016/j.pharmthera.2009.10.010

[48] Kroeze WK , Hufeisen SJ , Popadak BA *et al* H1‐histamine receptor affinity predicts short‐term weight gain for typical and atypical antipsychotic drugs. Neuropsychopharmacology 2003; 28: 519–526. 12629531 10.1038/sj.npp.1300027

[49] Wu C , Yuen J , Boyda HN *et al* An evaluation of the effects of the novel antipsychotic drug lurasidone on glucose tolerance and insulin resistance: A comparison with olanzapine. PLoS One 2014; 9: e107116. 25254366 10.1371/journal.pone.0107116 PMC4177840

[50] Srisawasdi P , Vanwong N , Hongkaew Y *et al* Impact of risperidone on leptin and insulin in children and adolescents with autistic spectrum disorders. Clin. Biochem. 2017; 50: 678–685. 28167244 10.1016/j.clinbiochem.2017.02.003

[51] Burghardt KJ , Seyoum B , Mallisho A , Burghardt PR , Kowluru RA , Yi Z . Atypical antipsychotics, insulin resistance and weight; A meta‐analysis of healthy volunteer studies. Prog. Neuropsychopharmacol. Biol. Psychiatry 2018; 83: 55–63. 29325867 10.1016/j.pnpbp.2018.01.004 PMC5817633

[52] Newcomer JW . Second‐generation (atypical) antipsychotics and metabolic effects: A comprehensive literature review. CNS Drugs 2005; 19: 1–93. 10.2165/00023210-200519001-00001 15998156

[53] Rummel‐Kluge C , Komossa K , Schwarz S *et al* Head‐to‐head comparisons of metabolic side effects of second generation antipsychotics in the treatment of schizophrenia: A systematic review and meta‐analysis. Schizophr. Res. 2010; 123: 225–233. 20692814 10.1016/j.schres.2010.07.012 PMC2957510

